# Plasma 1-deoxysphingolipids are early predictors of incident type 2 diabetes mellitus

**DOI:** 10.1371/journal.pone.0175776

**Published:** 2017-05-04

**Authors:** J. Mwinyi, A. Boström, I. Fehrer, A. Othman, G. Waeber, H. Marti-Soler, P. Vollenweider, P. Marques-Vidal, H. B. Schiöth, A. von Eckardstein, T. Hornemann

**Affiliations:** 1 Department of Neuroscience, Functional Pharmacology, Uppsala University, Uppsala, Sweden; 2 Institute of Clinical Chemistry, University and University Hospital of Zurich, Zurich, Switzerland; 3 Department of Medicine, Lausanne University Hospital (CHUV) and Faculty of Biology and Medicine, University Lausanne, Lausanne, Switzerland; Medical University of South Carolina, UNITED STATES

## Abstract

1-Deoxysphingolipids (1-deoxySLs) are atypical sphingolipids, which are formed in a side reaction during sphingolipid de-novo synthesis. Recently, we demonstrated that 1-deoxySLs are biomarkers for the prediction of T2DM in obese, non-diabetic patients. Here we investigated the relevance of 1-deoxySLs as long-term predictive biomarkers for the incidence of T2DM in an asymptomatic population. Here, we analyzed the plasma sphingoid base profile in a nested group of non-diabetic individuals (N = 605) selected from a population-based study including 5 year follow-up data (CoLaus study). 1-DeoxySLs at baseline were significantly elevated in individuals who developed T2DM during the follow-up (p<0.001), together with increased glucose (p<5.11E-14), triglycerides (p<0.001) and HOMA-IR indices (p<0.001). 1-Deoxy-sphinganine (1-deoxySA) and 1-deoxy-sphingosine (1-deoxySO) were predictive for T2DM, even after adjusting for fasting glucose levels in the binary regression analyses. The predictive value of the combined markers 1-deoxySA+glucose were superior to glucose alone in normal-weight subjects (p<0.001) but decreased substantially with increasing BMI. Instead, plasma adiponectin and waist-to-hip ratio appeared to be better risk predictors for obese individuals (BMI>30kg/m^2^). In conclusion, elevated plasma 1-deoxySL levels are strong and independent risk predictors of future T2DM, especially for non-obese individuals in the general population.

## Introduction

Obesity and diabetes mellitus type 2 (T2DM) are two strongly interrelated chronic diseases with dramatically increasing incidence worldwide [1]. Overweight and obesity are postulated to induce insulin resistance. Over several years, this prediabetic state progresses to manifest T2DM when insulin production does not suffice to compensate for insulin resistance. Asymptomatic for a long time, latent and manifest T2DM seed and promote the development of macro- and microvascular complications as well as peripheral neuropathy, long before clinical decompensation of hyperglycemia. Effective prevention of T2DM and its complications hence generate an urgent need of reliable and sensitive biomarkers for the early detection of individuals at high risk to develop T2DM [2, 3].

Sphingolipids are a heterogeneous class of lipid molecules typically composed on the eighteen carbon aliphatic amino-alcohol sphingosine [4]. Sphingolipid synthesis is initiated in the endoplasmic reticulum (ER) by serine palmitoyltransferase (SPT) which catalyzes the condensation of L-serine and palmitoyl-CoA (palm-CoA) to C18-Sphinganine (SA). The subsequent N-acylation to (dihydro)-ceramides forms the basis for the further transformation to complex sphingolipids such as sphingomyelins or glycosphingolipids. Several studies suggest that ceramides and other sphingolipids play a direct role in reducing insulin sensitivity (as reviewed by Chavez and Summers [5]). Atypical sphingolipids are synthesized by SPT through the use of acyl-CoAs other than palm-CoA or amino acids other than L-serine. In particular, the alternative use of L-alanine over L-serine forms an atypical class of 1-deoxysphingolipids (1-deoxySLs) which lack the C_1_-OH group of canonical sphingolipids. This renders 1-deoxySLs resistant to normal SL catabolism as the essential catabolic intermediate sphingosine-1-phosphate (S1P) cannot be formed. 1-DeoxySL are neurotoxic and pathologically elevated levels cause the inherited neuropathy HSAN1 (Hereditary Sensory Neuropathy Type I), which is associated with several missense mutations in the SPT genes. Also the wildtype SPT is able to form 1-deoxySLs to a certain extend and significantly elevated levels are found in plasma of individuals with metabolic syndrome (MetS) and T2DM [6, 7]. In this situation, the elevation is likely caused by an altered carbohydrate and fatty acid metabolism. Physiologically, the de-novo sphingolipid synthesis is located at a metabolic cross point which interconnects lipid metabolism with glucogenic amino acids and thereby also carbohydrate metabolism. At least in vitro, 1-deoxySL promote death of pancreatic beta-cells, interfere with insulin secretion [8] and contribute to the pathology of the diabetic sensory neuropathy [9, 10]. By prospective follow-ups of initially non-diabetic CVD patients we reported previously that 1-deoxySLs are associated with incident T2DM independently of glycated hemoglobin and MetS [7]. Despite the rather small number of events, this previous study suggested a promising potential of 1-deoxySLs as prospective biomarkers to identify individuals at high risk. Therefore, we aimed to further investigate the predictive value of deoxySLs in a larger and population based cohort also considering important covariates such as BMI and clinical measures of glycemia and dyslipidemia.

## Methods

### Study design

We included individuals from the CoLaus study, which has been described previously [11]. In brief, a non-stratified random sample of 35% of the Lausanne inhabitants aged 35–75 years (n = 56,694) was drawn. Individuals were recruited between June 2003 and May 2006, including 6,733 participants with a participation rate of 41%. All study participants were contacted for a follow-up investigation five years later, between 2009 and 2012, whereby examinations and procedures were performed in the same manner as during baseline investigation. All examinations and blood sampling procedures at baseline and follow-up were performed at the University Hospital of Lausanne after an overnight fast. Subjects were asked to refrain from heavy exercise and to maintain their usual diet the day before testing. Prescribed and non-prescribed drugs were reported and documented. Body weight and height measurements were performed in light clothes and without shoes. A non-stretchable tape was used to measure waist circumference twice on the unclothed abdomen at the narrowest point between the lower ribs and iliac crest, recording the mean value. Blood pressure was measured three times in a sitting position, in the left arm and after 10 minutes of relaxation. The study was conducted according to the principles expressed in the Declaration of Helsinki and approved by the Institutional Ethics Committee of the University of Lausann. Written informed consent was obtained from all subjects.

A nested group of 699 patients from the CoLaus study was selected. Incident T2DM cases and controls (ratio 1:2) were collected and matched for gender, age (±1 year) and BMI (±0.5 kg/m2). Inclusion criterion was the absence of T2DM at baseline as assessed by history and measurement of fasting plasma glucose < 7 mmol/L. Subjects with anti-diabetic treatment (insulin and/or oral drugs) and/or fasting plasma glucose levels ≥ 7mmol/L were classified as having T2DM. Based on these criteria, we excluded 94 subjects from the further analysis: 90 probands withT2DM at baseline and 4 lacking follow-up data for T2DM, resulting in a sample size of 605 individuals.

### Diagnosis of type 2 diabetes mellitus and metabolic syndrome

Diabetes mellitus at baseline was diagnosed according to the WHO criteria, i.e. fasting glucose levels ≥ 7 mmol/l (126 mg/dl) or a previous diagnosis of diabetes by a physician. Patients with fasting glucose levels between 5.9 and 6.9 mmol/L (100–125 mg/dl) were categorized as IFG. Individuals were classified as non-incident T2DM individuals when fasting glucose were within the physiological ranges at both baseline and follow-up surveys.

The diagnosis of MetS was based on the criteria of the National Cholesterol Education Program-Adult Treatment Panel III. Accordingly, at least three of the following criteria had to be met: waist circumference > 102 cm in men or >88 cm in women, triglycerides (TGs) ≥ 1.7 mmol/L (150 mg/dl), high-density lipoprotein (HDL) cholesterol < 1.0 mmol/L (40 mg/dl) in men or <1.3mmol/L (50 mg/dl) in women, blood pressure ≥ 130/≥85 mmHg, and fasting glucose ≥6.1 mmol/L (110 mg/dl).

For subsequent analyses we further subdivided individuals in a ‘Lean’ (BMI ≤ 27 kg/m^2^) and an ‘Obese’ (BMI ≥ 30 kg/m^2^) group.

### Determination of laboratory parameters

Blood samples were analyzed at the Clinical Laboratory of the Lausanne University hospital as described previously [11]. Clinical chemical assays were performed on fresh blood samples within 2 hours after collection.

### Sphingolipid analysis

Sphingoid bases in plasma are usually conjugated to a variety of N-linked fatty acids and different O-linked head groups. As we were interested in the total sphingoid base profile, we performed an acid/base hydrolysis prior to analysis to quantify the total amount of long chain bases. Therefore it is to note that, the here reported sphingoid base concentrations do not reflect the effective free sphingoid base levels in plasma but refer to the total sphingoid base composition of all plasma sphingolipids (independent whether free or N-acylated). Lipids were extracted from CoLaus EDTA plasma samples which had been stored at -80°C and analyzed as described previously [12]. Briefly, 0.5 ml methanol including 200 pmol of the internal standards d7-sphingosine and d7-sphinganine (d7SA, d7SO; Avanti Polar Lipids, Alabaster, Alabama, USA) was added to 100μl of plasma and extracted for 1h under agitation on a thermo-mixer at 37°C. Precipitated proteins were pelleted by centrifugation and the supernatant transferred to a new tube. For lipid hydrolysis, 75μl of methanolic HCl (1 N HCl and 10 M H2O in methanol) was added to the supernatant and incubated for 16 hours at 65°C. £Subsequently, 100 μl of 10M KOH was added to neutralize the HCl. To this mix 625 μl chloroform, 100 μl ammonium hydroxide (2N) and finally 0.5 ml alkaline water were added to complete the phase separation. The mix was vortexed and centrifuged at 16000 g for 5 minutes. After centrifugation, the upper phase was discarded and the lower organic phase washed three times with alkaline water (pH 10.3). Finally, the organic phase was dried under N2. Sphingoid bases were separated on a C18 column (Uptispere 120 Å, 5μm, 125 × 2 mm, Interchim, Montluçon, France) and analyzed on a TSQ Quantum Ultra mass spec (Thermo, Reinach, BL, Switzerland). Each sample was measured as a singleton. Intra- and inter-assay coefficient of variation (CV %) of the method was between 5% and 20%.

### Statistical analysis

Subjects were first divided into individuals with non-incident or incident T2DM, depending on their T2DM-status at follow-up investigations. Clinical variables and plasma sphingolipid levels were compared between the two groups by independent samples t-tests. A χ2 test was used to compare categorical variables. Furthermore, the predictive value of glucose, triglycerides, 1-deoxySA, 1-deoxySO and 1-deoxySL for the development of T2DM was tested in stepwise logistic regression analyses. Clinical variables and sphingolipid levels were log transformed prior to analysis, and all models were adjusted for age, gender, BMI and intake of lipid lowering drugs.

Multiple linear regression analyses were performed to investigate associations between the homeostatic assessment model values (HOMA-IR) and plasma levels of the sphingoid bases C18SA, C18SO, 1-deoxySO and 1-deoxySA, respectively. Both baseline and follow-up values of HOMA-IR were studied, and all models were adjusted for age, gender, BMI and the intake of lipid lowering drugs. For the analysis of baseline HOMA-IR indices, we also controlled for the presence of MetS. To further evaluate if BMI has an impact on the performance of glucose, TG and 1-deoxySA to predict incident T2DM, we performed logistic regression models in different weight categories, i.e. a) BMI ≤ 25 kg/m^2^, b) BMI ≤ 27 kg/m^2^ and c) BMI ≤ 30 kg/m^2^, adjusting for age, gender, BMI and intake of lipid-lowering drugs.

Furthermore, we compared log transformed clinical variables and plasma sphingolipids in non-incident and incident T2DM individuals in the ‘lean’ and ‘obese’ subgroup. The χ2 test was used to compare categorical variables. To further investigate the impact of BMI on the predictive value of different parameters, ROC curve analyses were performed to compare the prognostic performance of various continuous variables and different combinations thereof. All models were adjusted for glucose, age, gender, BMI and intake of lipid lowering drugs. Statistical analyses were performed in Excel, Analyze-IT (Analyse-it Software, Ltd., Leeds, UK), SPSS v.21.0 (IBM, Zurich, Switzerland) and R version 3.2.1 (R Foundation for Statistical Computing, www.R-project.org). NRI was calculated using the R-Script “PredicABEL” v1.2–2

## Results

### Plasma 1-deoxySA levels are significantly elevated in non-diabetic individuals who later on develop T2DM

The potential of plasma sphingoid bases to predict the risk for T2DM was first assessed by comparing the baseline levels of typical and atypical sphingoid bases between individuals who developed T2DM during the follow-up period (incident T2DM, n = 251) and those who did not (no incident T2DM, n = 354). As shown in **Table 1,** baseline 1-deoxySA was significantly higher in the group with incident T2DM, as were triglycerides, glucose and HOMA-IR indices (p<0.001 for 1-deoxySA, p<0.001 for triglycerides, p<5.11E-14 for glucose and p<0.001 for the HOMA-IR) in unadjusted analyses.

**Table 1:** Baseline values of clinical parameters and sphingolipids levels in the incident and non-incident T2DM group.

	No T2DM at follow-up (n = 354)	T2DM at follow-up (n = 251)	p value
Age (years)	56.9 (9.4)	56.9 (9.8)	0.906
Sex (female)	122 (34.5%)	87 (34.7%)	-
BMI (kg/m^2^)	28.72 (4.08)	29.03 (4.16)	0.367
Waist circumference (cm)	97.4 (11.5)	99.7 (11.4)	0.011
Waist-to-hip ratio	0.92 (0.08)	0.93 (0.07)	0.005
Cholesterol (mmol/L)	5.76 (1.07)	5.82 (1.02)	0.417
LDL (mmol/L)	3.54 (0.92)	3.53 (0.86)	0.967
HDL (mmol/L)	1.49 (0.38)	1.45 (0.39)	0.118
**Triglycerides (mmol/L)**	1.67 (1.15)	1.99 (1.77)	**0.001**
**Glucose (mmol/L)**	5.79 (0.53)	6.13 (0.51)	**5.11E-14**
Insulin (μU/mL)	10.53 (7.10)	12.11 (7.2)	0.011
**HOMA-IR**	2.74 (1.88)	3.28 (2.03)	**0.001**
Systolic BP (mm Hg)	135 (16)	136 (19)	0.519
Diastolic BP (mm Hg)	83 (10)	83 (12)	0.663
Adiponectin (mg/l)	8.59 (5.25)	7.52 (5.00)	0.007
Leptin (ng/ml)	15.31 (12.26)	15.29 (12.84)	0.923
ALT (U/l)	32.85 (19.85)	36.4 (21.33)	0.015
AST (U/l)	31.96 (11.42)	34.48 (19.07)	0.104
GGT (U/l)	37.43 (34.41)	44.34 (37.24)	0.002
CRP (mg/l)	85.04 (17.87)	81.93 (15.26)	0.029
*Plasma sphingolipids (μmol/L)*			
C16SO	17.67 (5.28)	17.7 (5.16)	0.924
C16SA	0.47 (0.18)	0.50 (0.21)	0.209
C17SO	8.41 (2.38)	8.20 (2.31)	0.238
C18PhytoSO	0.14 (0.04)	0.14 (0.04)	0.439
C18SAdiene	30.16 (7.43)	30.38 (7.25)	0.654
C18SO	90.42 (16.98)	91.66 (17.06)	0.359
C18SA	3.25 (1.00)	3.49 (1.22)	0.020
SLtotal	93.67 (17.59)	95.15 (17.75)	0.295
C19SO	2.32 (0.90)	2.29 (0.89)	0.592
C20SO	0.19 (0.05)	0.19 (0.05)	0.405
C20SA	0.02 (0.01)	0.02 (0.01)	0.026
1-deoxySO	0.16 (0.09)	0.18 (0.10)	0.008
**1-deoxySA**	0.08 (0.04)	0.09 (0.04)	**0.001**
deoxySLtotal	0.24 (0.12)	0.27 (0.14)	0.004

Values are shown as mean (SD) for the continuous variables and numbers and percent of total for the categorical variables. P-values were calculated using an unpaired two-sided t test on the log-transformed continuous variables. For the categorical variables, the p value was calculated using the χ2 test. Variables in bold show significant differences after Bonferroni correction (p<0.001). BMI, body mass index; CRP, C reactive protein

Other sphingoid bases such as C_18_SA, C_20_SA, 1-deoxySO were also elevated in the T2DM group, but did not reach statistical significance after correction for multiple comparisons. Correlation analysis (**S1 Table**) revealed a significant positive correlation between 1-deoxySL and TG (R = 0.53–0.55) whereas C_18_SA and C_18_SO correlated more with cholesterol levels (total, HDL-C, LDL-C).

### 1-deoxySA, 1-deoxySO and 1-deoxySL are significant independent predictors of T2DM, even after adjustment for glucose

Multiple linear regression analysis further showed significant correlations of C_18_SO, 1-deoxySA and 1-deoxySO with HOMA-IR after adjusting for age, gender, BMI, intake of lipid lowering drugs and presence of metabolic syndrome (**Table 2**). Interestingly, baseline plasma levels of 1-deoxySA and 1-deoxySO were also predictive of HOMA-IR indices at follow-ups.

**Table 2:** Multiple linear regression models of HOMA_IR at baseline and follow-up.

** **	Parameter	Beta	p Value
**Baseline**			
**Model 1**	**C**_**18**_**SO**	**-0.39**	**0.001**
**Model 2**	C_18_SA	0.08	0.223
**Model 3**	**1-deoxySO**	**0.12**	**0.007**
**Model 4**	**1-deoxySA**	**0.12**	**0.012**
**Follow-up**			
**Model 1**	C_18_SO	0.01	0.970
**Model 2**	**1-deoxySO**	**0.14**	**0.006**
**Model 3**	**1-deoxySA**	**0.17**	**0.005**

Stepwise logistic regression analysis (**Table 3A**) revealed that baseline 1-deoxySL, i.e. 1-deoxySO, 1-deoxySA are independent risk factors for the development of T2DM, even when adjusting for fasting glucose (p<0.006 for 1-deoxySA, p<0.032 for 1-deoxySO, p<0.018 for 1-deoxySL). However, none of these parameters were significant when also adjusting for triglycerides (TG).

**Table 3:** Binary logistic regression models for incident T2DM (n = 605).

**A)**			
**Incident Diabetes (overall)**	**Parameter**	**Beta**	**p Value**
**Model 1**	**Glucose**	**17.6**	**7.33E-13**
**Model 2**	**TG**	**1.2**	**0.002**
**Model 3**	**1-deoxySO**	**1.02**	**0.008**
**Model 4**	**1-deoxySA**	**1.54**	**0.001**
**Model 5**	**1-deoxySL**	**1.22**	**0.004**
**Model 6**	**Glucose**	**17.34**	**1.95E-12**
** **	**1-deoxySO**	**0.85**	**0.034**
**Model 7**	**Glucose**	**17.22**	**3.04E-12**
** **	**1-deoxySA**	**1.32**	**0.007**
**Model 8**	**Glucose**	**17.32**	**2.15E-12**
** **	**1-deoxySL**	**1.04**	**0.019**
**Model 9**	**Glucose**	**17.42**	**1.84E-12**
** **	**TG**	**1.12**	**0.006**
**Model 10**	**Glucose**	**17.32**	**2.60E-12**
** **	TG	0.85	0.078
** **	1-deoxySL	0.53	0.312
**B)**			
**Incident Diabetes (according to BMI)**	**Parameter**	**Beta**	**p Value**
**Model 1 (BMI < = 25, n = 99)**	**glucose**	**20.48**	**0.005**
** **	**1-deoxySA**	**3.66**	**0.009**
**Model 2 (BMI < = 27, n = 203)**	**glucose**	**17.61**	**2.29E-04**
** **	**1-deoxySA**	**2.47**	**0.005**
**Model 3 (BMI < = 30, n = 406)**	**glucose**	**14.56**	**1.31E-06**
** **	**1-deoxySA**	**1.47**	**0.0165**
**Model 4 (BMI < = 25, n = 99)**	**glucose**	**20.52**	**0.005**
** **	TG	0.36	0.797
** **	**1-deoxySA**	**3.41**	**0.043**
**Model 5 (BMI < = 27, n = 203)**	**glucose**	**18.26**	**1.66E-04**
** **	TG	1.14	0.194
** **	1-deoxySA	1.84	0.065
**Model 6 (BMI < = 30, n = 406)**	**glucose**	**14.62**	**1.31E-06**
** **	TG	0.69	0.205
** **	1-deoxySA	1.07	0.121

All models were adjusted for age, gender, BMI, lipid lowering drugs (y/n). Models at baseline were also adjusted for the metabolic syndrome (y/n). All variables were log transformed. Variables in bold are statistically significant (p<0.05). SA, sphinganine; SO, sphingosine; TG, triglycerides

## The predictive value of 1-deoxySL for incident T2DM is higher in normal-weight subjects

To investigate to what extent BMI contributes to the predictive value of sphingolipids in T2DM, we performed binary logistic regression analyses with incident T2DM as outcome variable in different BMI groups (BMI ≤25, BMI ≤27 and BMI ≤30 kg/m^2^). Interestingly, as shown in **Table 3B,** the relative contribution of glucose and 1-deoxySA in predicting T2DM, as evaluated by the regression β-value, decreased upon the inclusion of subjects with higher BMI. For the BMI ≤25 kg/m^2^ group 1-deoxySA remained predictive even after adjusting for TGs whereas significance is lost in the higher BMI groups. The interrelation of the predictive value and BMI is also reflected by changes of the area under the curve (AUC) in the ROC curve analyses for baseline glucose, triglycerides, 1-deoxySO, 1-deoxySA, 1-deoxySL, alone or in combination for different BMI cut-offs **(S2 Table)**. Here, the combined relative contribution of glucose and 1-deoxySA to the AUC decreased with increasing BMI cut-off levels (**S1 Fig**). Instead, glucose appeared to become more dominant in predicting T2DM in obese individuals. This was also reflected by the net reclassification improvement (NRI) which showed a significantly improvement for the combined markers glucose + 1-deoxySA in the BMI ≤25 group (p = 0.00711; NRI = 0.2048; [95% CI] 0.0557–0.3539) but not for the BMI ≤30 group (p = 0.1077).

To further investigate the predictive value of atypical sphingoid bases for incident T2DM in dependency of weight, we subdivided the cohort into two groups according to BMI, i.e. in ‘Lean’ (BMI<27 kg/m^2^) and ‘Obese’ individuals (BMI>30kg/m^2^). In the ‘Lean’ group (n = 203), we unraveled 1-deoxySA (p<0.001) and glucose as significant predictors of incident T2DM (**Table 4**). In contrast, only glucose (p<0.001) significantly predicted T2DM development in the ‘Obese’ group (n = 199). Binary logistic regression analyses using incident T2DM as outcome variable, revealed that for the ‘Obese’ group adiponectin and waist-hip-ratio are better predictors for incident T2DM than 1-deoxySA (**Table 5**). This observation is also supported by the ROC analyses, showing that adiponectin and waist-hip ratio are superior to 1-deoxySA in predicting incident T2D in combination with glucose in obese subjects (**S3 Table**).

**Table 4:** Baseline values of clinical variables and sphingolipids levels in the lean-sick and healthy obese group, comparing individuals with incident and non-incident T2DM after 5 years.

	Lean (n = 203)			Obese (n = 199)		
	No T2DM at follow-up (n = 122)	T2DM at follow-up (n = 81)	p Value (Baseline)	No T2DM at follow-up (n = 111)	T2DM at follow-up (n = 88)	p Value (Baseline)
Age (years)	56.24 (9.51)	57.17 (10.32)	0.579	56.31 (9.05)	57.13 (8.83)	0.519
Sex (female (%))	42 (34.42)	27 (33.33)	*-*	53 (47.75)	40 (45.45)	-
BMI	24.70 (1.64)	24.74 (1.63)	0.849	33.47 (2.97)	33.54 (2.96)	0.876
Waist circumference (cm)	87.83 (8.71)	89.62 (7.93)	0.126	106.64 (9.04)	109.81 (9.25)	0.016
**Waist-to-hip ratio**	0.88 (0.07)	0.90 (0.07)	0.076	0.93 (0.07)	0.96 (0.06)	0.004
Cholesterol (mmol/L)	5.66 (1.01)	5.76 (1.03)	0.509	5.86 (1.15)	5.84 (1.13)	0.922
LDL (mmol/L)	3.46 (0.95)	3.30 (1.09)	0.564	3.62 (0.97)	3.53 (0.91)	0.478
HDL (mmol/L)	1.58 (0.40)	1.61 (0.42)	0.698	1.41 (0.39)	1.36 (0.33)	0.286
Triglycerides (mmol/L)	1.36 (0.83)	1.87 (2.51)	0.024	1.81 (0.86)	2.18 (1.50)	0.047
**Glucose (mmol/L)**	5.81 (0.50)	6.11 (0.51)	**5. 82E-05**		5.74 (0.54)	6.20 (0.51)	**3.19E-09**
Insulin (μU/mL)	7.12 (4.36)	7.59 (5.79)	0.728	13.32 (6.10)	15.21 (8.07)	0.071
HOMA-IR	2.08 (1.10)	2.37 (1.52)	0.373	3.42 (1.65)	4.21 (2.29)	0.008
Systolic BP	131.59 (15.75)	134.80 (18.79)	0.239	133.98 (14.17)	140.25 (18.70)	0.010
Diastolic BP	81.17 (9.96)	80.59 (12.05)	0.603	83.69 (10.04)	86.86 (11.20)	0.040
Adiponectin (mg/l)	9.18 (4.67)	8.65 (5.52)	0.160	8.55 (4.89)	6.72 (4.48)	0.007
Leptin (ng/ml)	9.51 (8.17)	9.25 (8.33)	0.795	24.04 (13.81)	22.23 (15.00)	0.476
ALT (U/l)	29.11 (15.09)	32.25 (25.01)	0.510	37.08 (27.13)	40.61 (21.14)	0.388
AST (U/l)	30.11 (10.66)	30.48 (11.60)	0.914	32.95 (12.20)	38.01 (22.79)	0.103
GGT (U/l)	32.99 (33.16)	43.02 (41.84)	0.080	37.48 (34.38)	51.97 (54.34)	0.097
CRP (mg/l)	82.26 (16.58)	80.76 (12.60)	0.349	83.42 (15.15)	81.25 (16.54)	0.288
*Plasma sphingolipids*						
C16SO (μmol/L)	17.44 (5.63)	18.23 (4.88)	0.164	18.09 (4.96)	17.89 (5.60)	0.781
C16SA (μmol/L)	0.45 (0.17)	0.47 (0.19)	0.352	0.50 (0.18)	0.53 (0.23)	0.350
C17SO (μmol/L)	8.54 (2.40)	8.60 (2.28)	0.819	8.49 (2.33)	8.06 (2.41)	0.290
C18PhytoSO (μmol/L)	0.15 (0.04)	0.15 (0.05)	0.992	0.14 (0.05)	0.13 (0.04)	0.311
C18SAdiene (μmol/L)	29.79 (6.80)	30.84 (7.60)	0.355	31.45 (8.35)	30.50 (8.07)	0.651
C18SO (μmol/L)	90.14 (16.32)	93.37 (18.97)	0.237	90.31 (18.38)	90.44 (17.08)	0.974
C18SA (μmol/L)	3.03 (0.85)	3.26 (1.14)	0.206	3.44 (1.04)	3.87 (1.40)	0.069
SLtotal (μmol/L)	93.18 (16.89)	96.63 (19.55)	0.218	93.74 (19.01)	94.31 (18.01)	0.878
C19SO (μmol/L)	2.36 (0.97)	2.33 (0.90)	0.966	2.33 (0.83)	2.19 (0.85)	0.559
C20SO (μmol/L)	0.19 (0.05)	0.19 (0.06)	0.758	0.19 (0.06)	0.20 (0.05)	0.653
C20SA (μmol/L)	0.02 (0.01)	0.02 (0.01)	0.222	0.02 (0.01)	0.03 (0.02)	0.018
1-deoxySO (μmol/L)	0.14 (0.07)	0.17 (0.12)	0.032	0.17 (0.09)	0.21 (0.14)	0.125
1-deoxySA (μmol/L)	0.07 (0.03)	0.09 (0.05)	**9.0E-04**		0.09 (0.04)	0.11 (0.06)	0.103
deoxySLtotal (μmol/L)	0.21 (0.10)	0.26 (0.16)	0.013		0.26 (0.13)	0.32 (0.20)	0.100

The ‘Lean‘-group includes subjects with a BMI equal to or less than 27 kg/m2. The ‘Obese’-group includes subjects with a BMI equal to or more than 30 kg/m2.

Values are shown as mean±SD for the continuous variables and numbers and per cent of total for the categorical variables. P-values were calculated using an unpaired two-sided t test on the log-transformed continuous variables. For the categorical variables, the p value was calculated using the χ2 test. Variables in bold font have significant differences after Bonferroni correction (p<0.001).

ALT, alanine transaminase; ASP, aspartate transaminase; BMI, body mass index; GGT, gamma-glutamyl transpeptidase; LDL-C, low-density lipoprotein cholesterol; HDL-C, high-density lipoprotein cholesterol; TG, triglycerides; HOMA-IR, homeostatic model assessment of insulin resistance; SA, sphinganine; SO, sphingosine; WHR, waist-hip ratio

**Table 5:** Binary logistic regression models for incident T2DM, participants with a BMI ≥ 30 kg/m^2^ (n = 199).

		Beta	p Value
**Model 1**	**glucose**	**22.53**	**2.56E-07**
	doxSA	1.19	0.154
**Model 2**	**glucose**	**24.00**	**2.24E-07**
	**adiponectin**	**-2.12**	**0.002**
**Model 3**	**glucose**	**22.17**	**6.37E-07**
	**waist-hip-ratio**	**18.46**	**0.009**

All models were adjusted for age, gender, BMI and intake of lipid lowering drugs. Variables are log transformed. Variables in bold are statistically significant (p<0.05). BMI, body mass index; SA, sphinganine; SO, sphingosine; TG, triglycerides

## Discussion

In two previous studies [9, 12], we demonstrated that plasma levels of 1-deoxySLs are significantly elevated in CVD patients with MetS and T2DM and predict the incidence of T2DM in initially non-diabetic CVD patients. In this prospective study of a general population, we confirmed and extended these findings. Unlike established biomarkers of increased risk for future diabetes, 1-deoxySLs showed best prognostic performance in normal weight individuals that do not have obesity as a classical risk factor for T2DM development. These findings indicate that 1-deoxySLs may aid in the identification of patients at increased risk for T2DM, especially if they do not fulfill the typical clinical criteria. Furthermore, this observation indicates a pathogenic role of atypical sphingolipids in the development of T2DM. Yet, only scarce information is available on both the metabolic basis of elevated 1-deoxySL plasma levels in prediabetes and the diabetogenic mechanisms exerted by 1-deoxySLs. The finding of elevated 1-deoxySL plasma levels appears to be rather specific for T2DM, as Wei et al. [13] found them typically elevated in patients with T2DM but less in patients with diabetes mellitus type 1 (T1DM). The authors also demonstrated that 1-deoxySL are not associated with hyperglycemia per se but rather with metabolic changes in T2DM notably hypertriglyceridemia [13]. In the present study as well as in our previous ones [9] we observed a significant correlations between plasma 1-deoxySLs and triglycerides. However, despite this high co-linearity, a stepwise logistic regression analysis revealed that triglycerides and 1-deoxySL predicted incident T2DM independently of each other [7]. This finding was also confirmed in the present study which showed that 1-deoxySA is an independent predictor for T2DM in lean individuals (BMI<25kg/m2) even when adjusting for both glucose and TG (**Table 2B—Model 4**). Interestingly, the predictive value of the 1-deoxySLs was generally higher in normal weight individuals than in obese subjects. At higher BMIs the contribution of fasting glucose and triglycerides became increasingly dominant. For individuals with BMI>30 deoxySLs were not significantly predictive anymore. Here adiponectin or waist-to-hip ratio were better predictors. The metabolic connection between plasma triglycerides and 1-deoxySLs is enigmatic as 1-deoxySL are formed by the alternate use of alanine over serine and not by a different fatty acid substrate. One explanation could be that deoxySLs are exclusively transported by VLDL and LDL lipoproteins [6, 7]. However, we previously showed that only the treatment with fenofibrate but not with nicotinic acid lowered plasma levels of 1-deoxySLs although both treatments lowered TG levels [14]. Another link may be non-alcoholic fatty liver disease (NAFLD) which is typically associated with hypertriglyceridemia. In fact, elevated plasma 1-deoxySLs were found to be associated with NAFLD and non-alcoholic steatohepatitis (NASH) [15]. A direct implication of deoxySLs in the pathogenesis of T2DM was suggested by data from in-vitro studies, showing that deoxySLs inhibit glucose-stimulated insulin secretion and induce cell death in both the rat insulinoma cell line Ins-1 and primary rodent islets [8]. The association of 1-deoxySLs with incident T2DM may have clinical implications not only for the early identification of patients at increased risk but also for the prevention of the yet underdiagnosed and undertreated diabetic distal sensorimotor polyneuropathy (DSPN). Originally associated with rare inherited sensory neuropathy (HSAN1) we found elevated 1-deoxySL plasma levels associated with DSPN. [10]. In a rat model of DSPN, neurological symptoms were prevented or improved after a pharmacological lowering of 1-deoxySLs [9].

The strengths of this study are the prospective design, the population-based set up and the considerable event rate enabling correction for many important confounders. A limitation is the nested case-control design which does not allow calculating general predictive values. It will be hence important to further validate our findings in larger cohorts and also in different ethnicities to establish the independent association between 1-deoxySL plasma levels and the risk of T2DM in general and specifically in non-obese individuals. Further research is needed to unravel the pathogenic link between atypical sphingolipids and T2DM.

In summary, we showed that increased plasma 1-deoxySL levels, especially of 1-deoxy-SA, are independent risk factors of T2DM development especially for non-obese individuals.

## Supporting information

S1 Table Pearson correlation coefficients for the analyzed sphingoid bases, clinical chemistry, and anthropometric variables. (PDF)

S2 Table Areas under the curves as measurement for the predictive value of different parameters for incident T2DM and stratified by BMI category. (PDF)

S3 Table Areas under the curves as measurement for the predictive value of different parameters for incident T2DM. (PDF)

S1 Fig AUC values for glucose and glucose + 1-deoxySA, obtained in ROC curve analyses at different arbitrary BMI cut-offs. (PDF)

S1 Data Raw data and models. (ZIP)

## Abbreviations

1-deoxySA: 1-deoxysphinganine
1-deoxySL: 1-deoxysphinoglipids
1-deoxySO: 1-deoxysphingosine
ALT: alanine aminotransferase
AST: aspartate aminotransferase
BP: blood pressure
BMI: body mass index
ER: endoplasmatic reticulum
GGT: gamma-glutamyltransferase
HDL: high density lipoprotein
HOMA-IR: homeostatic model assessment
HSAN1: hereditary sensory neuropathy type I
IFG: impaired fasting glucose
LDL: low density lipoprotein
MetS: metabolic syndrome
palmitoyl CoA: palmitoyl coenzyme A
SPT: serine-palmitoyltransferase
TG: triglycerides
T2DM: type 2 diabetes mellitus
