# Correction: Plasma 1-deoxysphingolipids are early predictors of incident type 2 diabetes mellitus

**DOI:** 10.1371/journal.pone.0179313

**Published:** 2017-06-05

**Authors:** J. Mwinyi, A. Boström, I. Fehrer, A. Othman, G. Waeber, H. Marti-Soler, P. Vollenweider, P. Marques-Vidal, H. B. Schiöth, A. von Eckardstein, T. Hornemann

The following information is missing from the Funding section: TH also received funding from Stiftung für wissenschaftliche Forschung, University Zurich.
