# Supplementary material for: Plasma 1-deoxysphingolipids are early predictors of incident type 2 diabetes mellitus
Source: PLoS One. 2017 May 4;12(5):e0175776. doi: 10.1371/journal.pone.0175776 (PMC5417440; doi:10.1371/journal.pone.0175776)
Supplement: S1 Table — (PDF) [file pone.0175776.s001.pdf]

S1 Table Pearson correlation coefficients for the analyzed sphingoid bases, clinical chemistry, and anthropometric variables

**Coef.**	**C_{16}SO**	**C_{16}SA**	**C_{17}SO**	**C_{18}PhytoSO**	**C_{18}SAdiene**	**C_{18}SO**	**C_{18}SA**	**SL total**	**C_{19}SO**	**C_{20}SO**	**C_{20}SA**	**deoxySO**	**deoxySA**	**deoxySL total**
**Age**	**0.17**	0.02	**0.18**	0.04	**0.18**	0.07	0.01	0.06	**0.15**	**0.10**	0.05	**0.15**	0.07	**0.13**
**BMI**	0.00	**0.10**	-0.06	**-0.19**	0.05	-0.07	**0.13**	-0.06	-0.03	-0.01	**0.10**	**0.13**	**0.15**	**0.14**
**Waist circumference**	-0.07	0.06	**-0.16**	**-0.21**	**-0.14**	**-0.12**	**0.12**	**-0.11**	-0.06	0.03	**0.12**	**0.27**	**0.24**	**0.27**
**WHR**	**-0.16**	0.00	**-0.26**	**-0.13**	**-0.30**	**-0.14**	0.08	**-0.12**	**-0.12**	0.05	**0.11**	**0.30**	**0.24**	**0.29**
**Cholesterol**	**0.55**	**0.46**	**0.51**	**0.48**	**0.55**	**0.72**	**0.56**	**0.73**	**0.27**	**0.43**	**0.29**	**0.36**	**0.44**	**0.39**
**LDL-C**	**0.45**	**0.40**	**0.47**	**0.45**	**0.47**	**0.68**	**0.51**	**0.69**	**0.27**	**0.42**	**0.24**	**0.20**	**0.28**	**0.23**
**HDL-C**	**0.38**	**0.12**	**0.36**	**0.20**	**0.47**	**0.39**	**0.13**	**0.38**	**0.15**	**0.15**	0.05	-0.06	-0.05	-0.06
**TG**	0.02	**0.13**	**-0.10**	0.04	**-0.09**	-0.06	**0.14**	-0.05	**-0.09**	0.00	**0.12**	**0.53**	**0.54**	**0.55**
**Glucose**	-0.04	0.04	**-0.12**	0.01	**-0.11**	-0.05	**0.08**	-0.04	**-0.12**	0.04	**0.11**	**0.15**	**0.13**	**0.14**
**Insulin**	-0.02	**0.08**	**-0.11**	**-0.16**	**-0.14**	**-0.19**	**0.09**	**-0.17**	-0.03	0.00	**0.09**	**0.20**	**0.19**	**0.20**
**HOMA-IR**	-0.03	**0.08**	**-0.12**	**-0.15**	**-0.15**	**-0.19**	**0.09**	**-0.17**	-0.05	0.00	**0.11**	**0.21**	**0.20**	**0.21**
**Systolic BP**	0.04	**0.09**	-0.01	0.08	0.01	0.04	**0.13**	0.04	-0.04	0.02	**0.13**	**0.17**	**0.15**	**0.17**
**Diastolic BP**	0.05	**0.14**	-0.02	0.00	-0.01	0.06	**0.18**	0.07	-0.06	0.04	**0.14**	**0.17**	**0.19**	**0.18**
**Adiponectin**	**0.22**	0.02	**0.23**	**0.09**	**0.32**	**0.16**	0.00	**0.15**	**0.12**	0.03	**-0.09**	**-0.12**	**-0.13**	**-0.13**
**Leptin**	**0.17**	**0.17**	**0.18**	**-0.09**	**0.25**	0.04	**0.16**	0.05	**0.14**	0.02	**0.10**	0.01	0.04	0.02
**ALT**	**-0.13**	0.05	**-0.23**	**-0.14**	**-0.25**	**-0.16**	**0.11**	**-0.15**	**-0.14**	0.00	**0.11**	**0.16**	**0.13**	**0.16**
**AST**	-0.06	**0.09**	**-0.18**	0.02	**-0.20**	**-0.10**	**0.18**	**-0.08**	**-0.14**	0.03	**0.12**	**0.23**	**0.22**	**0.24**
**GGT**	**-0.10**	0.07	**-0.24**	-0.06	**-0.24**	**-0.09**	**0.16**	-0.08	**-0.16**	0.00	**0.15**	**0.32**	**0.27**	**0.32**
**CR**	-0.05	-0.03	-0.07	-0.06	**-0.16**	-0.06	-0.02	-0.06	-0.04	0.04	-0.01	**0.16**	**0.13**	**0.15**

ALT, alanine transaminase; ASP, aspartate transaminase; BMI, body mass index; GGT, gamma-glutamyl transpeptidase; LDL-C, low-density lipoprotein cholesterol; HDL-C, high-density lipoprotein cholesterol; TG, triglycerides; HOMA-IR, homeostatic model assessment of insulin resistance; SA, sphinganine; SO, sphingosine; WHR, waist-hip ratio. Statistically significant associations are indicated in bold
