# Supplementary material for: Plasma 1-deoxysphingolipids are early predictors of incident type 2 diabetes mellitus
Source: PLoS One. 2017 May 4;12(5):e0175776. doi: 10.1371/journal.pone.0175776 (PMC5417440; doi:10.1371/journal.pone.0175776)
Supplement: S2 Table — (PDF) [file pone.0175776.s002.pdf]

S2 Table Areas under the curves as measurement for the predictive value of different parameters for incident T2DM, stratified by BMI category

BMI cut-off	Glucose	doxSA	Glucose + doxSA	delta AUC (Glucose *vs* Glucose + doxSA)
≤25	0.690	0.644	0.740	0.050
≤26	0.694	0.636	0.736	0.041
≤27	0.685	0.620	0.712	0.028
≤28	0.657	0.602	0.676	0.019
≤29	0.654	0.583	0.671	0.018
≤30	0.649	0.579	0.666	0.016
≤31	0.661	0.574	0.670	0.009
≤33	0.660	0.582	0.673	0.013
≤35	0.670	0.581	0.679	0.009

All models adjusted for glucose, TG, doxSO, doxSA, doxSL, glucose + doxSO, glucose + doxSA, glucose + doxSL, glucose + TG, glucose + TG + doxSL.
