# Supplementary material for: Plasma 1-deoxysphingolipids are early predictors of incident type 2 diabetes mellitus
Source: PLoS One. 2017 May 4;12(5):e0175776. doi: 10.1371/journal.pone.0175776 (PMC5417440; doi:10.1371/journal.pone.0175776)
Supplement: S3 Table — (PDF) [file pone.0175776.s003.pdf]

S4 Table. Areas under the curves as measurement for the predictive value of different parameters for incident T2DM, participants with a BMI ≥ 30 kg/m^2

Parameter	Area	Std. Error	p-value
**Glucose**	0.781	0.031	9.41E-13
**doxSA**	0.620	0.038	0.002
**Waist-hip-ratio**	0.627	0.038	0.001
**Glucose + doxSA**	0.785	0.031	4.67E-13
**Glucose + adiponectin**	0.802	0.029	1.78E-14
**Glucose + waist-hip ratio**	0.795	0.030	6.26E-14
