# Supplementary material for: Plasma 1-deoxysphingolipids are early predictors of incident type 2 diabetes mellitus
Source: PLoS One. 2017 May 4;12(5):e0175776. doi: 10.1371/journal.pone.0175776 (PMC5417440; doi:10.1371/journal.pone.0175776)
Supplement: S1 Fig — (PDF) [file pone.0175776.s004.pdf]

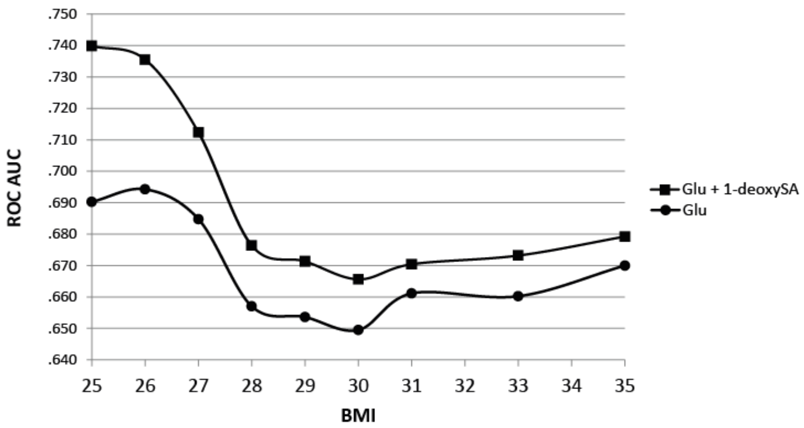

S3 Figure AUC values for glucose (blue) and glucose + 1-deoxySA (green), obtained in ROC curve analyses at different arbitrary BMI cut-offs. The AUC for incident T2DM of glucose + 1-deoxySA (green) is larger than that of glucose alone, especially at low BMI values < 27, and converges at higher BMIs.
